# *Daucus carota* pentane-based fractions arrest the cell cycle and increase apoptosis in MDA-MB-231 breast cancer cells

**DOI:** 10.1186/1472-6882-14-387

**Published:** 2014-10-10

**Authors:** Wassim N Shebaby, Mohammad Mroueh, Kikki Bodman-Smith, Anthony Mansour, Robin I Taleb, Costantine F Daher, Mirvat El-Sibai

**Affiliations:** Department of Microbial and Cellular Sciences, Faculty of Health and Medical Sciences, University of Surrey, Surrey, UK; School of Pharmacy, Lebanese American University, P.O. Box 36, Byblos, Lebanon; School of Medicine, Lebanese American University, Byblos, Lebanon; Department of Natural Sciences, School of Arts and Sciences, Lebanese American University, P.O. Box 36, Byblos, Lebanon

**Keywords:** Daucus carota, Breast cancer, Proliferation, Apoptosis, PI3K, MAPK

## Abstract

**Background:**

*Daucus carota* L.ssp.carota (wild carrot), an herb used in folk medicine worldwide, was recently demonstrated to exhibit anticancer activity. In this study we examined the anticancer effect of Daucus carota oil extract (DCOE) fractions on the human breast adenocarcinoma cell lines MDA-MB-231 and MCF-7 and clarified the mechanism of action.

**Methods and results:**

Using the WST assay, the pentane fraction (F1) and 1:1 pentane:diethyl ether fraction (F2) were shown to possess the highest cytotoxicity against both cell lines. Flow cytometric analysis revealed that both fractions induced the accumulation of cells in the sub-G1 phase, increase in apoptotic cell death and chromatin condensation. The increase in apoptosis in response to treatment was also apparent in the increase in BAX and the decrease in Bcl-2 levels as well as the proteolytic cleavage of both caspase-3 and PARP as revealed by Western blot. Furthermore, treatment of MDA-MB-231 cells with either fraction significantly reduced the level of phosphorylated Erk but did not show any effect on phosphorylated Akt. The combined treatment with a potent PI3K inhibitor (wortmannin) and F1 or F2 fraction had a synergistic inhibitory effect on cell survival which shows that these two drugs work on different pathways.

**Conclusions:**

These results suggest that *the pentane-based fractions of DCOE possess potential anti-cancer activity that is mainly mediated through the Erk pathway.*

**Electronic supplementary material:**

The online version of this article (doi:10.1186/1472-6882-14-387) contains supplementary material, which is available to authorized users.

## Background

Breast cancer is one of the most common cancers among women aged between 40–55 years, and is the leading cause of death in women globally [1]. The present treatment approaches, whether surgery, chemotherapy, immunotherapy or radiotherapy are usually accompanied with adverse effects, leaving the patient weak and vulnerable. Furthermore, acquired patient resistance to these treatments poses a continuing problem that compromises the effectiveness of anti-cancer therapy [2]. Therefore, there is a growing interest in finding more effective and selective anti-cancer remedies. The use of natural therapeutic compounds of plant origin has been of interest to researchers for the past few decades. In fact, over 25% of pharmaceutical drugs used in medicine today are derived from plants [3]. For instance, plant secondary metabolites such as terpenes, phenolics and alkaloids, are extensively exploited in cancer research [4–9]. Currently, there are four classes of plant-derived anticancer compounds in clinical use: vinca alkaloids, epipodophyllotoxins, taxanes, and camptothecin derivatives [10].

Among the hallmarks of cancer cells are aberrant cell cycle and apoptosis regulation. Cancer cells are known to lose the ability to negatively regulate the cell cycle leading to their continuous proliferation [11] and also resist apoptosis even in the presence of apoptotic stimuli [12]. Accordingly, a wide range of phytochemicals derived from medicinal plants have been reported to exhibit anticancer activity by targeting several signaling cascades associated with cell cycle regulation and apoptosis. These cascades include mitogenic signaling pathways, such as mitogen-activated protein kinases (MAPKs) [13], PI3K-Akt pathway [14] and nuclear kappa B pathway (NF-kB) [15]. These alterations induced by phytochemicals are eventually correlated to the modulation of cell cycle and/or apoptosis of cancer cells [16].

Wild carrot, *Daucus carota* L. ssp. carota, is a spiny–fruited herb that grows in moderate regions throughout the world. The *Daucus carota* oil extract from various geographical locations constitutes mainly of monoterpenes, sesquiterpenes, and phenylpropanoids [17, 18]. Unlike the edible carrot, *Daucus carota* L. ssp. sativus, few reports exist about the potential therapeutic use of the wild carrot. In European folk medicine, it is used as a urinary antiseptic and anti-inflammatory remedy for cystitis and prostatitis [19]. The plant has also been reported to possess antilithic, diuretic, [20, 21] antibacterial, and antifungal activities [18, 22, 23]. Recent studies conducted in our laboratories, showed that *Daucus carota* oil extract (DCOE) exhibited anti-tumor [24, 25], antioxidant [24], anti-inflammatory, and anti-ulcer [26] activities. The present study aims to evaluate the anticancer activity of DCOE fractions against MDA-MB-231 and MCF-7 human breast cancer cell lines and to elucidate possible mechanisms of action.

## Methods

### Reagents

Dulbecco’s modified Eagle’s medium (DMEM) and dimethyl sulfoxide (DMSO) were purchased from Sigma (St. Louis, USA). The Annexin V/PI apoptosis detection kit was purchased from Abcam (Cambridge, UK), and WST-1 reagent was purchased from Roche (Mannheim, Germany). All other chemicals used in this study were purchased from Sigma (St. Louis, USA) unless otherwise stated.

### Sample collection and oil extraction

*Daucus carota* (Linnaeus) ssp. carota mature umbels were collected at the post flowering season between May and August from Byblos, Lebanon. The plant was identified according to the characteristics described in the “Handbook of Medicinal Herbs” [21] and confirmed by Dr. A. Houri, a Lebanese plant expert at the Lebanese American University. A voucher specimen of the plant material used in this study has been deposited in a publicly available herbarium. The extraction procedure was carried out according to the method described by Zeinab et al. [25]. Briefly, umbels were air dried in the shade and then cut into small pieces for oil extraction in methanol/acetone (1:1) for 72 h. The extract was then filtered and evaporated to dryness under reduced pressure. The residue was centrifuged and the oil was dried over anhydrous sodium sulfate. The final yield (3.47%) was stored in a closed amber bottle at 4°C until use.

### DCOE fractionation

Thirty grams of *DCOE* were chromatographed on a silica gel column (35–70 mesh). The first fraction (F1) was eluted with pentane (100%), the second fraction (F2) with pentane: diethyl ether (50:50), the third fraction (F3) with diethyl ether (100%) and the fourth fraction (F4) with chloroform: methanol (93:7). Fractions were analyzed by TLC using hexane: ethyl acetate (70:30) as mobile phase and plates were stained with 2% anisaldehyde.

### Cell lines and culture

Human breast adenocarcinoma cell lines MDA-MB-231 and MCF-7 were purchased from American Type Culture Collection (ATCC, Rockville). Both cell lines were cultured in a humidified incubator at 37°C and 5% CO_2_ atmosphere in DMEM (Dulbecco’s modified Eagle’s medium), supplemented with 10% fetal bovine serum and 1% Penicillin-streptomycin.

### Cell proliferation assay

The proliferation of the MDA-MB-231 and MCF-7 cells was tested using WST-1 assay. Cells were plated in 96-well plates at a concentration of 10^5^ cell/ml for 24 h. Both cell lines were then treated with increasing concentrations (10, 25, 50, and 100 μg/ml) of the four DCOE fractions in DMSO for 48 h. At the end of the treatment period, WST-1 reagent was added to the cells and incubated in a humidified incubator at 37°C and 5% CO_2_ atmosphere for 3 h. The intensity of the produced formazan was quantified at 450 nm using a microplate ELISA reader. For wortmannin treatment, MDA-MB-231 cells were incubated with or without wortmannin (1 μM) for 1 h in a serum-free complete MEM prior to treating cells with 25 and 50 μg/ml of F1 and F2 fractions for 48 h.

### Apoptosis assay

The apoptotic effect of the most potent fractions F1 and F2 of DCOE on MDA-MB-231 cells was determined by Annexin V-FITC staining assay and measured by C6 flow cytometer (BD Accuri Cytometers, Ann Arbor, MI USA). The MDA-MB-231 cells (1 × 10^5^ cells/ml) were treated with different concentrations (25 and 50 μg/ml) of both fractions and cultured in 6-well plates for 48 h. Treated cells were harvested, washed with phosphate-buffer saline (PBS) and then centrifuged at 500 g for 5 min. Then cell pellet was suspended in 500 μL of Annexin V-FITC/PI apoptosis detection kit at room temperature for 5 min in the dark. Annexin V-FITC binding was analyzed by flow cytometry (Ex = 488 nm; Em = 530 nm) FL1 channel for detecting Annexin V-FITC staining and FL3 channel for detecting PI staining. Annexin V-positive, PI-negative cells were scored as early apoptotic, and double-stained cells were considered as late apoptotic.

### DAPI and annexin V staining

MDA-MB-231 cells were seeded on 25 mm square glass cover slips. After treatment, cells were washed with PBS and fixed with 3.7% paraformaldehyde for 10 min at room temperature. Fixed cells were permeabilized with a 0.1% solution of Triton X-100 in PBS and then stained by Annexin V conjugated to FITC or with 1 μg/ml of DAPI solution for 20 min. The cells were washed with PBS and observed with fluorescence microscope.

### Cell cycle analysis

The effect of F1 and F2 fractions on cell cycle distribution was assessed by flow cytometry after staining the MDA-MB-231 cells with propidium iodide (PI). Briefly, the MDA-MB-231 cells (1 × 10^5^ cells/ml) were treated with different concentrations (25 and 50 μg/ml) of both fractions for 48 h. The treated cells were harvested, washed with PBS and fixed with 70% ethanol on ice. Then cells washed with cold PBS, suspended in 200 μL 1X Propidium Iodide + RNase staining solution and incubate at 37°C in the dark for 30 minutes. Propidium Iodide Flow Cytometry Kit was used for cell cycle analysis. DNA content of the cells was measured by C6 flow cytometer and the population of each phase was determined using CFlow Plus analysis software (BD Accuri Cytometers, Ann Arbor, MI USA).

### Western blot

MDA-MB-231 cells were treated with different concentrations of F1 and F2 (25 and 50 μg/ml) for 48 h. The adherent and non-adherent MDA-MB-231 cells were collected on ice, washed twice with PBS, lysed with lysis buffer, and centrifuged at 12,000 g for 10 min at 4°C. The cell lysate was heated at 100°C for 5 min, and the protein content was determined by the Bio-Rad protein assay (Bio-Rad, Hercules, CA, USA). The same amount of proteins was loaded to a 10% SDS-PAGE. Proteins were then transferred to PVDF membrane (Pall Corporation, Ann Arbor, USA) and blocked with 5% skim milk for 2 h. The membranes were probed with primary antibodies against Actin, p53, Bcl-2, Bax, Caspase-3, PARP, Akt, p-Akt, Erk, and p-Erk (Abcam, Cambridge, USA) at 4°C overnight. Later, the primary antibodies were washed away with TBST for 1 h and the membranes were treated with HRP-coupled secondary antibodies (Promega Corp., Madison, USA) for 1 h, and washed with TBST afterwards. Finally, Detection of each protein was performed using the ECL kit (Abcam plc, 330 Cambridge Science Park, Cambridge UK).

### Statistical analysis

Data was analyzed for statistical significance using one way analysis of variance (ANOVA). Values of the different tested parameters within each group are presented as mean ± SEM. Significant main effect differences were tested using Bonferroni post hoc test for multiple comparisons. All data were analyzed with the statistical package SPSS 18, and differences between groups were considered statistically significant if p < 0.05.

All experimental protocols were approved by the Animal Ethical Committee of the Lebanese American University, which complies with the Guide for the Care and Use of Laboratory Animals (Committee for the Update of the Guide for the Care and Use of Laboratory Animals, 2010).

## Results

**Figure 1:**
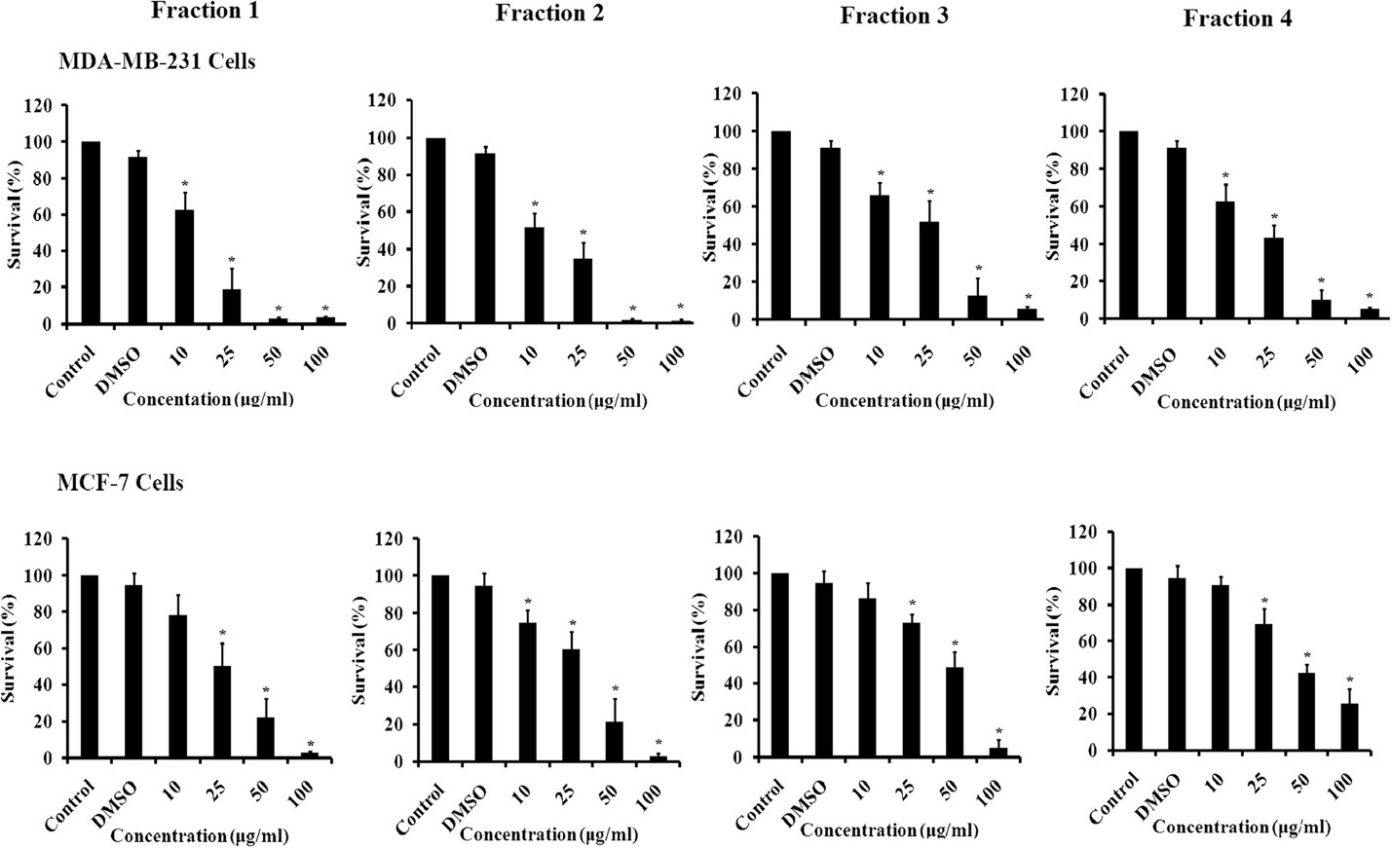
**Effect of DCOE fractions on cell proliferation.** MDA-MB-231 and MCF-7 cells were treated with DCOE fractions (F1, F2, F3, and F4) at concentrations of 10, 25, 50, and 100 μg/ml or with 0.5% DMSO for 48 h. Data are expressed as% survival of cells relative to control. Data are the mean ± SEM from three independent experiments. *denotes P < 0.05 versus DMSO group as measured by one-way ANOVA.

MDA-MB-231 and MCF-7 breast cancer cells were treated with different concentrations of fractions 1, 2, 3, and 4 of DCOE for 48 h (Figure 1). The treatment with the extract appeared to be dose-dependent with maximum inhibition of proliferation at higher concentrations (50 and 100 μg/ml). MCF-7 cells showed the least responsiveness to the extract when compared to its more malignant counterpart the MDA-MB-231 cell line. The IC_50_ values of the four fractions at 48 h were 22, 32, 48, and 43 μg/ml for MCF-7 and 17, 11, 27, and 23 μg/ml for MDA-MB-231. The pentane fraction (F1) and pentane/diethyl fraction (F2) showed the highest cytotoxicity against both cell lines with the lowest IC_50_ (Additional file 1: Table S1).

**Figure 2:**
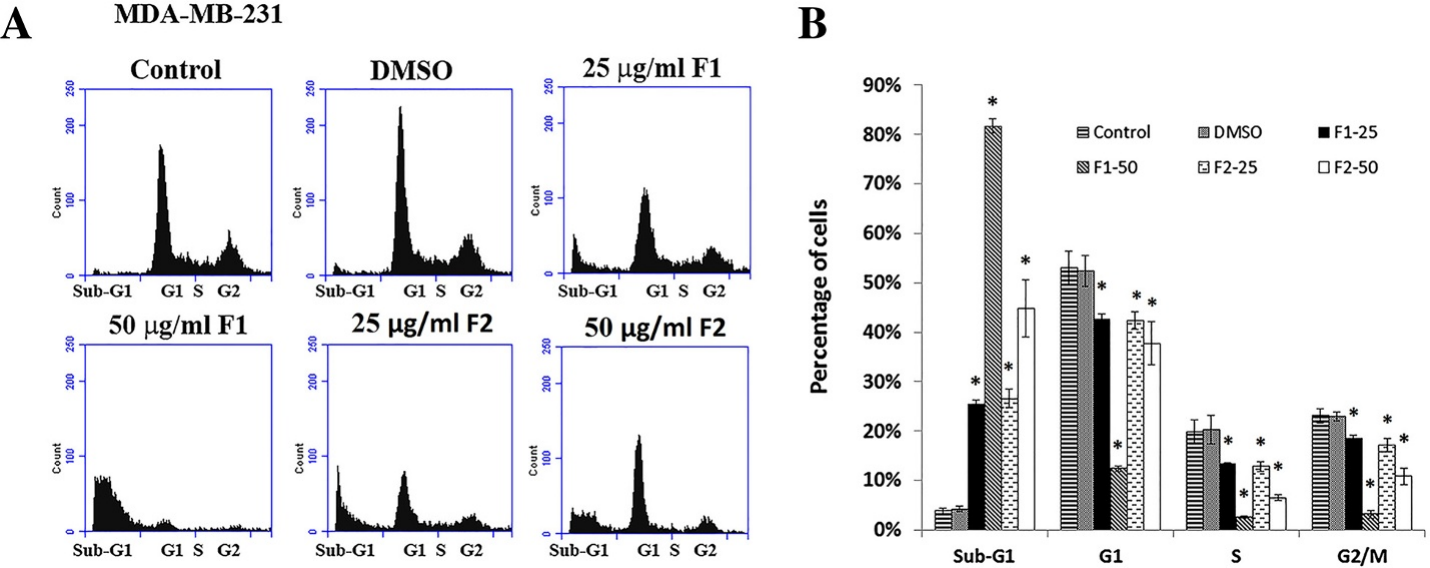
**Effect of F1 and F2 fractions on the cell cycle distribution in MDA**-**MB**-**231. (A)** MDA-MB-231 cells were treated with 25 and 50 μg/ml of F1 or F2 fractions and control cells were treated with 0.5% DMSO for 48 hours, after which cells were stained with PI and analyzed for DNA content by flow cytometry. The sub-G1 peak is considered as the apoptotic portion. The results shown are representative of 3 independent experiments. **(B)** Bar graph shows the cell distributions of each phase of the cell cycle. Data are means ± SEM of three independent experiments. *denotes P < 0.05 versus DMSO group as measured by one-way ANOVA.

**Figure 3:**
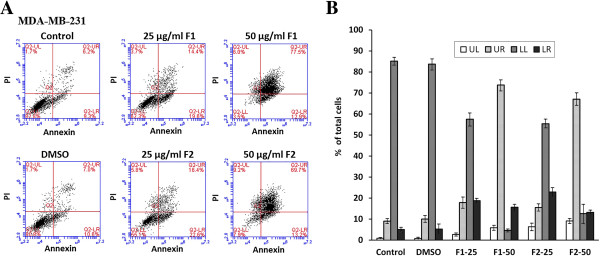
**Apoptotic effect of F1 and F2 treatment on MDA**-**MB**-**231 cells. (A)** Flow cytometric analysis of Annexin V-FITC and propidium iodide (PI) quantifying the F1 and F2-induced apoptosis in MDA-MB-231. Dot plots of MDA-MB-231 cells treated with 0.5% DMSO, 25 or 50 μg/ml of F1or F2 fractions for 48 hours. Viable cells are in the lower left (LL) region (negative for both annexin V-FITC and PI). Early apoptotic cells are located in the lower right (LR) region (annexin V-FITC positive). Late apoptotic cells demonstrating extensive cellular and nuclear membrane damage are located in the upper right (UR) region (double positive). Necrotic cells with destroyed cell membrane are in the upper left (UL) region (PI positive). The results shown are representative of three independent experiments. **(B)** Bar graphs showing the percentages of cell population of each quadrant in non-treated and treated MDA-MB-231 cells. Data are means ± SEM of three independent experiments.

**Figure 4:**
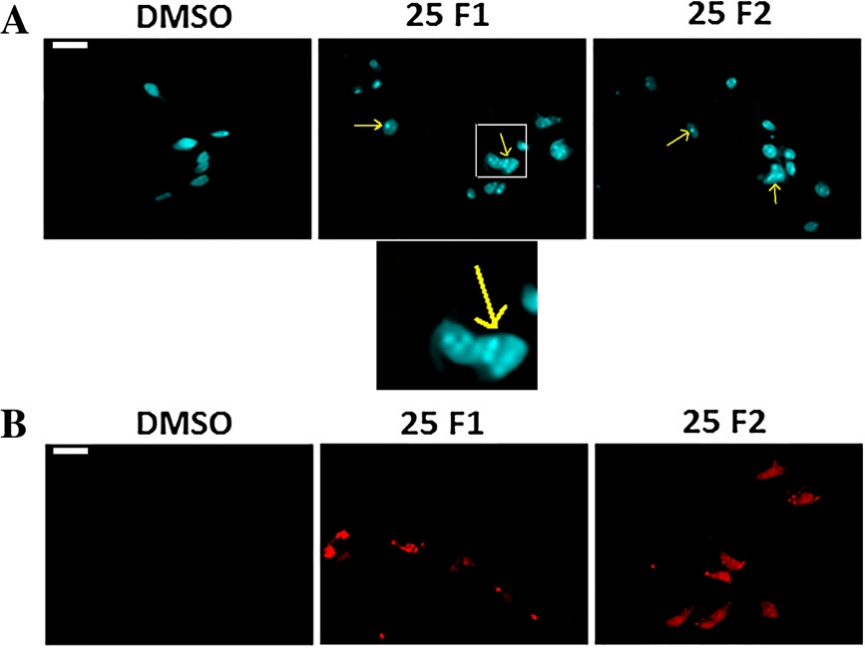
**Apoptotic morphological changes of MDA**-**MB**-**231 cells following treatment with fractions F1 or F2.** Fluorescent micrographs of control (0.5% DMSO) and MDA-MB-231cells treated with 25 μg/ml of F1 or F2 fractions for 48 hours and stained with DAPI **(A)** or annexin **(B)** and examined by fluorescence microscopy. Arrows in **A** indicate apoptotic bodies of nuclear fragmentation. Scale bar is 10 μm.

To determine whether growth inhibition by F1 and F2 on MDA-MB-231 human breast cancer cells was due to cell cycle arrest or apoptosis, the cells were stained with PI after permeabilization to look at the cell cycle profile or with Annexin and PI to look for apoptosis. As shown in Figure 2, treatment of MDA-MB-231 with F1 and F2 (25 and 50 μg/ml) for 48 h resulted in the increase of cell percentage in the sub-G1 phase with hypodiploid nuclei, indicating that both fractions could cause DNA fragmentation. This increase was coupled with the decreased percentage of cells in the S and G2/M phases. Figure 3 showed that cells treated with 25 μg/ml F1 or F2 were in early apoptosis whereas cells treated with 50 μg/ml went into late apoptosis. To further confirm the apoptotic effect of F1 and F2, control and treated MDA-MB-231 cells were stained with DAPI and annexin. Condensation of chromatin in nuclei was evident in cells treated with either dose of F1 (Figure 4A). Besides, annexin staining showed that both fractions induced the phosphatidylserine (PS) translocation in the cell membrane which is indicative of apoptosis followed by membrane leakage (Figure 4B).

**Figure 5:**
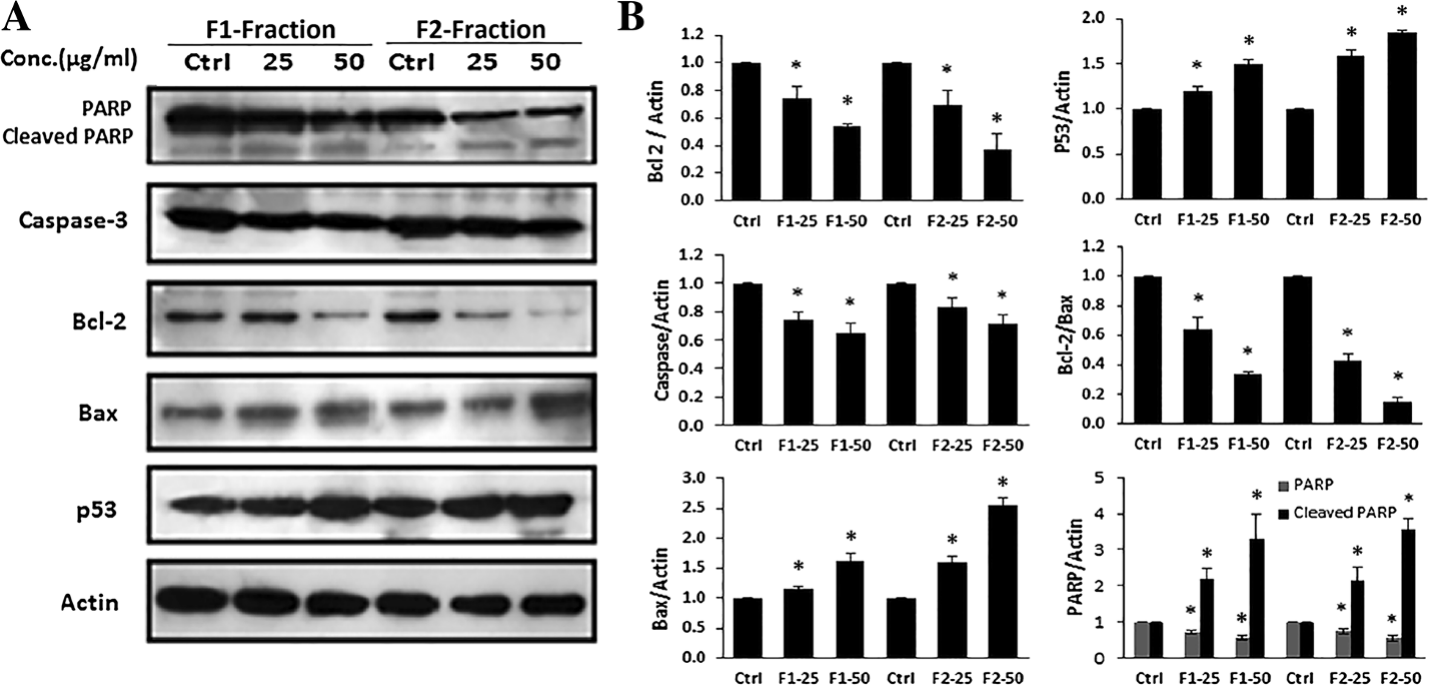
**Western blot analysis of apoptosis related proteins in MDA**-**MB**-**231 cells. (A)** Effect of F1 and F2 fractions on the expression of PARP, p53, caspase-3, Bcl-2, and Bcl2-associated X (BAX) proteins. Expression of β-actin was used as an internal control. MDA-MB-231 cells were treated with 25 and 50 μg/ml of F1 or F2 fractions and control cells were treated with 0.5% DMSO for 48 hours. Western blots are representative of three independent experiments. **(B)** The densitometer-intensity data of the proteins of each blot is presented as mean ± SEM from three independent experiments. *denote P < 0.05 versus control (0.5% DMSO) as measured by one-way ANOVA.

To explore the mechanism by which F1 and F2 induced apoptosis, the expression levels of various pro-apoptotic and anti-apoptotic proteins was evaluated by western blot. Treatment of MDA-MB-231 cells with F1 and F2 induced a decrease in the level of the inactive caspase-3 in a dose dependent manner indicating its cleavage into activated form. In response to treatment, the level of the 116-kDa PARP decreased and the level of the cleaved 85-kDa fragment increased (Figure 5A). Furthermore, the results of western blot indicated that the level of the anti-apoptotic protein Bcl-2 was decreased, whereas the pro-apoptotic protein BAX was increased. The expression of p53 protein was also assessed by western blot and showed an increased level as shown in Figure 5A.

**Figure 6:**
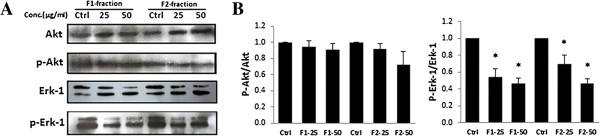
**Western blot analysis of PI3K**/**Akt and MAPK**/**Erk pathways in MDA**-**MB**-**231 cells. (A)** Effect of F1 or F2 fractions on the expression of Akt, p-Akt, Erk, p-Erk proteins in MDA-MB-231 cells. Cells were treated with 25 and 50 μg/ml of F1 or F2-fractions and control cells treated with 0.5% DMSO for 48 hours. Western blots were representative of three independent experiments. **(B)** The densitometer-intensity data of the proteins of each blot is presented as mean ± SEM from three independent experiments. *denotes P < 0.05 versus control (0.5% DMSO) as measured by one-way ANOVA.

PI3K activation was examined using western blot analysis to look for phosphorylated Akt. Upon treatment of MDA-MB-231 with either fraction, no significant change in phosphorylated Akt was observed as compared with the control (Figure 6). On the other hand, the activity of the MAPK pathway was assessed through examining the level of phosphorylation of Erk. Results in Figure 6 revealed a significant decrease in pErk upon treatment with F1 or F2.

**Figure 7:**
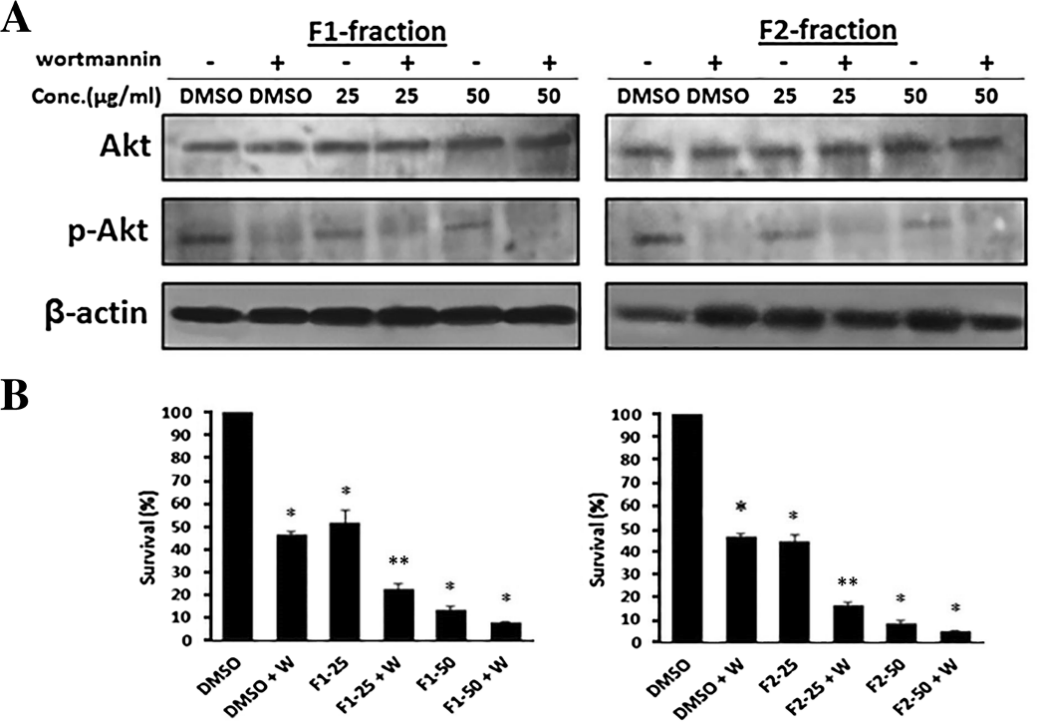
**Effect of the combined treatment of F1 or F2 with wortmannin on MDA**-**MB**-**231 cells. (A)** MDA-MB-231 cells were treated with 25 or 50 μg/ml of F1 or F2 or DMSO for 48 in the presence or absence of 1 μM wortmannin for 24 h. Cells were lysed and blotted for Akt and pAkt. **(B)** Effect of F1 and F2 fractions on MDA-MB-231 cell survival at concentrations of 25 or 50 μg/ml for 48 hours in the presence or absence 1 μM wortmannin. Inhibition of cell survival was assessed using the WST-1 assay. Data are expressed as% survival of cells. Data denote mean ± SEM from three independent experiments. *denotes P < 0.05 versus control (0.5% DMSO) and **denotes P < 0.05 versus F1-25 or to F2-25 groups as measured by one-way ANOVA.

Since neither fraction acted through the PI3K pathway in MDA-MB-231 cells, we investigated whether the combined treatment of each fraction with wortmannin, a selective PI3K inhibitor, would have a synergistic effect on the inhibition of cell proliferation. WST results showed a substantial decrease in cell survival of MDA-MB-231 cells treated with 25 μg/ml of F1 or F2 along with wortmannin (1 μM) compared to fraction alone (Figure 7A and B).

## Discussion

DCOE has been shown to have an antioxidant and anticancer activities against breast and colon cancer cell lines [24]. Previously, DCOE has been fractionated into four major fractions and various constituents of each fraction were identified and their *in vitro* and *in vivo* antioxidant activities were evaluated (Shebaby, et al. 2013, submitted). The present data demonstrated that the DCOE fractions had significant anti-proliferative effect against MDA-MB-231 and MCF-7 human breast cancer cell lines. The DCOE fractions exhibited dose dependent cytotoxicity against both cell lines. Moreover, pentane (F1) and pentane/diethyl ether (F2) fractions exhibited higher anticancer activity than the other fractions (F3, F4).

This anticancer activity might be attributed to major components present in the oil fractions. For example, *α*-humulene and *β*-caryophyllene which are major components of F1, have been reported to exhibit cytotoxic activity against several human cell lines [27, 28]. Similarly, the sesquiterpene *β*-selinene and the phenylpropanoids, (*E*)-methylisoeugenol and elemicin, identified in F2 fraction are also known to demonstrate anti-proliferative activity and tumoricidial effects against several human cell lines [8]. The anti-proliferative activity of the F2 fraction may be attributed to the presence of the major compound which is tentatively identified *via* GC-MS as 2-himachalene-6-ol. To the best of our knowledge, this compound has not been reported in any of the wild carrot species around the world and there are no previous studies involving its anticancer activity. However, widdrol, a structural isomer of 2-himachalen-6-ol, extracted from *Juniperus chinensis*, has been shown to exhibit a potent anti-cancer activity against colon adenocarcinoma cells HT29 by inhibiting their proliferation and inducing cell cycle G1 arrest [29]. In another study, widdrol induced G1 arrest through the induction of Chk2, p53 phosphorylation and CDK inhibitor p21 expression as well as inhibition of cyclin E, cyclin-dependent kinase (CDK2) and retinoblastoma protein (pRB) [30].

Targeting the cell cycle and apoptotic pathways in cancer cells is an important approach for cancer treatment and anti-cancer drug development [31]. Therefore, many studies have been conducted to isolate and identify compounds that induce cell cycle arrest and apoptosis [32]. In this study, we found that fractions F1 and F2 induced apoptosis in MDA-MB-231 cells, as verified by the formation of apoptotic nuclei that are characterized by chromatin condensation and DNA fragmentation. These results are consistent with an accumulation of cells in the sub-G1 apoptotic phase. The cell cycle analysis also revealed a decrease in cycling cells, which is indicative of cell cycle arrest. The tumor suppressor p53 plays an important role in response to DNA damage or other genomic instability. Functional p53 protein is crucial in p53-dependent pathway leading to cell cycle arrest or apoptosis [33]. MDA-MB-231 cells are known to harbor high level of mutant and non-functional form of p53 [34]. The increase in p53 protein following F1 or F2 treatment may not solely explain p53-dependent apoptosis. Therefore, the cell cycle arrest and apoptosis in MDA-MB-231 cells is chiefly mediated through a p53-independent mechanism.

To gain an understanding into the mechanisms controlling apoptosis induced by F1 and F2, we examined several components of the apoptotic pathways. The proteins of the Bcl-2 family play an essential role in apoptosis and are considered as a target for anticancer therapy [35, 36]. The Bcl-2 protein exhibits an anti-apoptotic effect while BAX is a pro-apoptotic protein of the Bcl-2 family [37, 38]. Several studies indicated that the ratio of Bcl-2 to BAX proteins decreases during apoptosis [39]. In the present study, a decrease in the expression Bcl-2 and an increase in the expression of BAX were observed after treating MDA-MB-231 cell lines with F1 and F2. Additionally, activated caspase 3 plays a crucial role in the final step of apoptosis and it is a key protease for the cleavage of PARP resulting in the loss of its enzymatic activity during apoptosis. This nuclear enzyme is involved in DNA repair and maintenance of genomic integrity and it has been used as an important marker of apoptosis [40]. The present data demonstrate that cleavage of caspase 3 and PARP are evident after treatment of MDA-MB-231 cells with F1 and F2 [41] which is consistent with an increase in apoptosis.

The MAPK/ERK pathway plays an important role in several cellular processes such as cell survival, cell proliferation and apoptosis. It is reported that constitutively activated ERK is responsible for high levels of cell growth in various cancers [42, 43]. On the other hand, down-regulation of ERK activity is highly associated with apoptosis [44]. In the present study, treatment of MDA-MB-231 cells with either F1 or F2 significantly decreased the phosphorylated ERK. Therefore, induction of apoptosis in MDA-MB-231 cells after F1 or F2 treatment may be mediated through the inhibition of the MAPK pathway.

The PI3K/AKT is another important intracellular signaling pathway in regulating cell proliferation and apoptosis. The serine/threonine protein kinase Akt is a downstream molecule which is involved in the inactivation of several pro-apoptotic proteins and the activation of anti-apoptotic proteins. The Akt pathway has an essential role in regulating cell proliferation and survival in cancer [45, 46]. Our results revealed that apoptotic concentrations of the F1 or F2 did not show any significant effect on phosphorylated Akt compared to the control. However, a combined treatment with a potent PI3K inhibitor (wortmannin) had a synergistic effect on apoptosis potentially through the inhibition of both MAPK and PI3K pathways. This shows that wortmannin and the fractions are acting distinct pathways.

## Conclusion

The present study demonstrates that the pentane fraction (F1) and pentane/diehtylether fraction (F2) have potent anticancer activity against MDA-MB-231 and MCF-7 human breast cancer cells. Both fractions inhibit cell proliferation by inducing cell cycle arrest and apoptosis in MDA-MB-231 cells. This induction of apoptosis is through the inhibition of the ERK pathway. The results suggest that the wild carrot could be considered a potential source for natural anticancer compounds. Future work will focus on isolation and characterization of the tentatively identified major compound and will explore the prospective *in vitro* and *in vivo* anticancer capabilities.

## Electronic supplementary material

Additional file 1: Table S1: IC_50_ values of the different fractions. (TIFF 29 KB)
