# Supplementary figures and images for: Daucus carota pentane-based fractions arrest the cell cycle and increase apoptosis in MDA-MB-231 breast cancer cells
Source: BMC Complement Altern Med. 2014 Oct 10;14:387. doi: 10.1186/1472-6882-14-387 (PMC4203935; doi:10.1186/1472-6882-14-387)

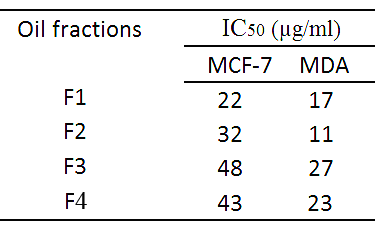

Supplement: Supplementary file 1 — Additional file 1: Table S1: IC50 values of the different fractions. (TIFF 29 KB) [file 12906_2014_1969_MOESM1_ESM.tiff]
